# Beyond Ethylene: New Insights Regarding the Role of Alternative Oxidase in the Respiratory Climacteric

**DOI:** 10.3389/fpls.2020.543958

**Published:** 2020-10-27

**Authors:** Seanna Hewitt, Amit Dhingra

**Affiliations:** ^1^ Molecular Plant Sciences Program, Washington State University, Pullman, WA, United States; ^2^ Department of Horticulture, Washington State University, Pullman, WA, United States

**Keywords:** alternative oxidase, fruit, ethylene, respiration, climacteric ripening, glyoxylic acid, tricarboxylic acid cycle, metabolism

## Abstract

Climacteric fruits are characterized by a dramatic increase in autocatalytic ethylene production that is accompanied by a spike in respiration at the onset of ripening. The change in the mode of ethylene production from autoinhibitory to autostimulatory is known as the System 1 (S1) to System 2 (S2) transition. Existing physiological models explain the basic and overarching genetic, hormonal, and transcriptional regulatory mechanisms governing the S1 to S2 transition of climacteric fruit. However, the links between ethylene and respiration, the two main factors that characterize the respiratory climacteric, have not been examined in detail at the molecular level. Results of recent studies indicate that the alternative oxidase (AOX) respiratory pathway may play an essential role in mediating cross-talk between ethylene response, carbon metabolism, ATP production, and ROS signaling during climacteric ripening. New genomic, metabolic, and epigenetic information sheds light on the interconnectedness of ripening metabolic pathways, necessitating an expansion of the current, ethylene-centric physiological models. Understanding points at which ripening responses can be manipulated may reveal key, species- and cultivar-specific targets for regulation of ripening, enabling superior strategies for reducing postharvest wastage.

## Introduction

Ripening of fruit involves a symphony of transcriptionally and hormonally controlled processes that result in the accumulation of sugars, reduction in acidity, development of aroma, and nutritional profiles (McAtee et al., 2013; Cherian et al., 2014). The ripening process has been under continual manipulation both as a result of natural selection for improved seed dispersal as well as human domestication via the selection of desirable organoleptic properties (Giovannoni, 2004; Liu M. et al., 2015).

**Figure 1:**
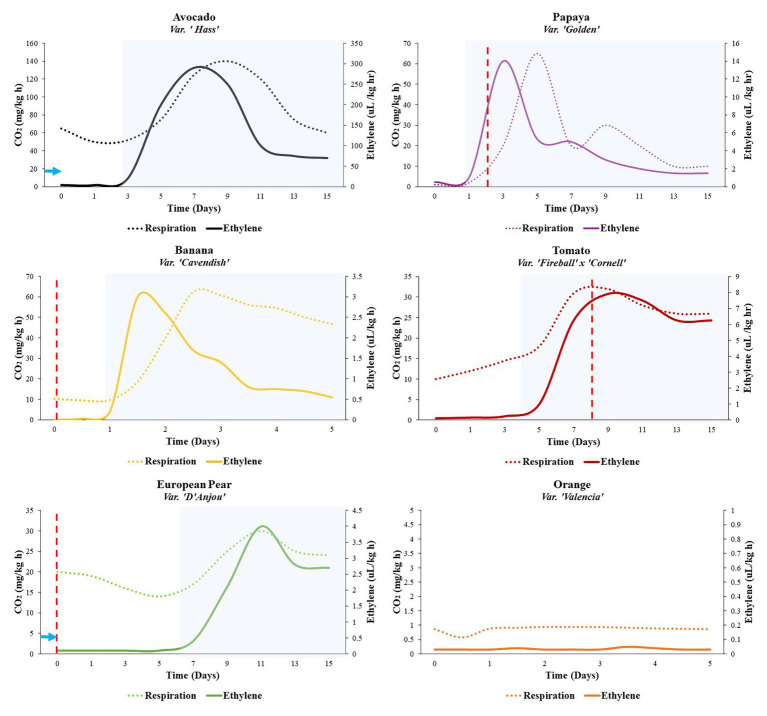
Ripening profiles of five climacteric fruits and one nonclimactic fruit. Rates of ethylene production and respiration are indicated by the solid and dotted lines, respectively. At the 0-day time point, fruits were unripe but assumed to have reached physiological maturity and 100% ripening competency. Some fruits require cold conditioning in order to achieve ripening competency (e.g., European pear, 15–90 days, 0–10°C) (solid blue arrow). Other fruits can be induced to ripen more quickly through appropriate cold storage (e.g., avocado, 14 days, 5–10°C; dashed blue arrow; Eaks, 1983). Expression and activity of alternative oxidase (AOX) have been implicated in preclimacteric and/or climacteric stages of some fruits (e.g., European pear, papaya, banana, and tomato; Woodward et al., 2009; Oliveira et al., 2015; Hendrickson et al., 2019; Hewitt et al., 2020b). Peak AOX expression, as indicated in the cited studies, is indicated by vertical dashed red lines. The transition from white to shaded background represents the transition from S1 to S2 ethylene (preclimacteric to climacteric shift). General respiration and ethylene profiles for each fruit were adapted from the following studies: avocado (Eaks, 1983), banana (McMurchie et al., 1972), European pear (Wang and Mellenthin, 1972), papaya (Fabi et al., 2007), tomato (Herner and Sink, 1973), orange (McMurchie et al., 1972).

Fleshy fruits fall into one of two broadly defined ripening categories, climacteric and nonclimacteric, based on their respiratory profile as well as the manner in which they produce the phytohormone ethylene (Seymour et al., 2012). Nonclimacteric fruits respire and produce ethylene at basal levels throughout fruit maturation and senescence. This mode of ethylene production is termed System 1 (S1) ethylene production. Nonclimacteric fruits, including cherries, berries, and citrus, are harvested ripe and do not exhibit increasing levels of ethylene production during ripening, although in a few cultivars, ripening may be accelerated through exogenous application of ethylene or ethylene-producing compounds, such as ethrel (Barry et al., 2000; Barry and Giovannoni, 2007; Chen et al., 2018). In contrast, ripening in climacteric fruits, such as apple, pear, banana, papaya, avocado, mango, and tomato, is characterized by a burst of respiration accompanied by a substantial increase in ethylene biosynthesis as fruit transitions from S1 to System 2 (S2) ethylene production (Figure 1; Osorio et al., 2013b; Chen et al., 2018). The synchronization of the hallmark respiratory rise with autocatalytic ethylene production forms the basis for the modern understanding of climacteric ripening (Lelièvre et al., 1997; Cara and Giovannoni, 2008). Because of this distinct ripening physiology, climacteric fruits can be harvested unripe and ripened off the tree or vine (Seymour et al., 2013a; Hiwasa-Tanase and Ezura, 2014). Following the respiratory climacteric, ripening proceeds rapidly and irreversibly, which presents additional challenges to the storage and preservation of climacteric fruit after harvest (Jogdand et al., 2017). While the concept of two distinct ripening categories is simple in theory, the reality is far more complex, with certain fruits displaying variable phenotypes (Paul et al., 2012). Interestingly, some cultivars within the same species display differences in ripening profiles; such is the case for, peach, plum, melon, and Chinese pear (Yamane et al., 2007; Minas et al., 2015; Saladié et al., 2015; Farcuh et al., 2018). This is clearly exemplified in Japanese plum (*Prunus salicina* Lindl.), where a nonclimacteric cultivar and an ethylene-responsive, suppressed-climacteric cultivar were both found to be derived from independent bud sport mutations in a single climacteric plum variety (Minas et al., 2015). This discovery suggests that the basis for the distinction between climacteric versus nonclimacteric ripening is highly specific at the genetic level. Furthermore, such systems provide natural models that could facilitate study of the biological basis for climacteric and nonclimacteric ripening (Kim H. -Y. et al., 2015; Minas et al., 2015; Fernandez i Marti et al., 2018).

Transcriptional and phytohormonal regulation of ethylene-dependent ripening have been reviewed extensively (Cherian et al., 2014; Karlova et al., 2014; Kumar et al., 2014; Chen et al., 2018). In contrast to climacteric fruit, the regulatory network involved in nonclimacteric ripening has been much less studied. Nevertheless, it is known that abscisic acid (ABA) and polyamines, rather than ethylene, play essential roles in ripening in nonclimacteric fruits (Li et al., 2011; Jia et al., 2016a). Studies in strawberry and tomato indicate that the split between climacteric and nonclimacteric ripening responses lies in the way that S-adenosyl-L-methionine (SAM) is preferentially utilized as a precursor to ethylene or as a substrate for polyamine biosynthesis (Van de Poel et al., 2013; Lasanajak et al., 2014; Guo et al., 2018). Decarboxylation of SAM by decarboxylase (SAMDC) represents the rate-limiting step in polyamine biosynthesis (Handa and Mattoo, 2010; Seymour et al., 2013b; Cherian et al., 2014). Moreover, transgenic expression of a yeast SAMDC in tomato results in preferential shunting of substrate into the polyamine biosynthesis pathway, rather than the ethylene biosynthesis pathway (Lasanajak et al., 2014). In addition to increased flux through the polyamine biosynthesis pathway, overexpression of SAMDC in strawberry leads to spermine and spermidine-mediated increase in expression of positive regulators of ABA biosynthesis and signaling (Guo et al., 2018). Signaling components downstream of ABA receptors are believed to induce changes in the expression of genes associated with pigment development and sugar metabolism (Li et al., 2011).

Through the exploration of the underlying genetic factors of ripening of both climacteric and nonclimacteric fruit in model systems, foundations have been laid for evaluation of ripening processes in nonmodel fruits exhibiting deviations from the standard profiles. Not surprisingly, manipulation of environmental factors, genetic factors, and use of chemical inhibitors like 1-methylcyclopropene (1-MCP) to inhibit ripening result in developmental patterns that do not follow the classical model of ethylene response and signaling (Watkins, 2006, 2015; Tatsuki et al., 2007; Chiriboga et al., 2013). Mechanisms for blockage and/or bypass of the concerted steps in classical ethylene biosynthesis are beginning to be elucidated as more studies examine how genetic manipulation or stimulation via temperature or chemical application affect ripening (Klee and Giovannoni, 2011; Hewitt et al., 2020a,b). Furthermore, as molecular biology, transcriptomics, and epigenetic analysis tools have rapidly advanced, new insights have been gained into some of the master regulators of ripening acting upstream and/or independently of ethylene (Liu R. et al., 2015; Giovannoni et al., 2017).

The alternative oxidase (AOX) respiratory pathway has recently garnered interest as a potential target for ripening manipulation (Hendrickson et al., 2019; Hewitt et al., 2020a,b). This review will explore AOX as a branch point for variation from the classical model of climacteric ripening, which may be affected by physiological or chemical perturbations in metabolism, transcriptional regulatory elements, and epigenetic signatures regulating fruit ripening. Understanding these variations is expected to inform novel strategies to reduce postharvest waste while improving marketability of fruit efficiently.

### Reexamining the Classical Model for Ethylene-Dependent Ripening

Early knowledge of the role of ethylene in the ripening process made components of ethylene biosynthesis and transduction some of the first targets for ripening control in model systems such as tomato (*Solanum lycopersicum*) (Barry and Giovannoni, 2007; Klee and Giovannoni, 2011; Liu M. et al., 2015; Mattoo and White, 2018). Resulting as a side product of methionine cycling (Yang and Hoffman, 1984), ethylene biosynthesis begins with the conversion of L-methionine into SAM. SAM is then converted into 5'-methylthioadenosine (MTA) and 1-aminocyclopropane carboxylate (ACC) via ACC synthase (ACS). ACC is subsequently converted into ethylene by 1-aminocyclopropane carboxylate oxidase (ACO). ACC, long known for its role as the immediate precursor to ethylene, has recently been investigated for its potential role in ethylene-independent regulation of growth (Polko and Kieber, 2019; Vanderstraeten et al., 2019). Following successful perception of ethylene, the hormone signal is transduced via a series of messengers to the nucleus where ethylene-responsive transcription factors (ERFs) activate downstream ripening-associated genes involved in cell wall softening, starch to sugar conversion, aroma production, and changes in pigmentation, among numerous other changes (Seymour et al., 2012; Osorio et al., 2013a; Cherian et al., 2014; Gao et al., 2020).

**Figure 2:**
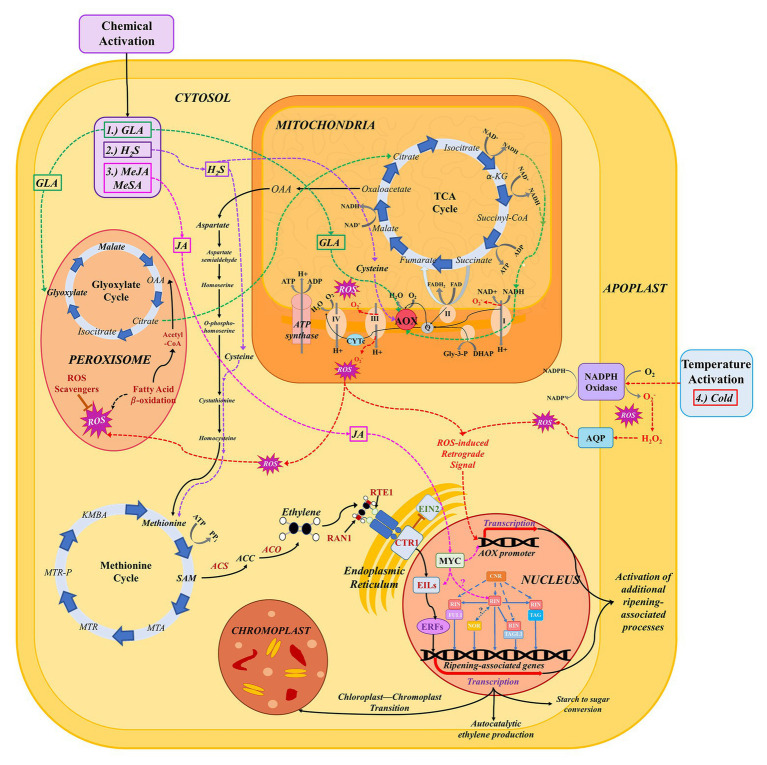
The network of pathways involved in respiration, ethylene biosynthesis, and signaling, and ROS production and signaling during the ripening process. Pathways implicated in the stimulation of AOX expression or activity are indicated by dashed, colored lines. (1) Glyoxylic acid (GLA; green): GLA has been shown to directly activate AOX *in vivo* via interaction with the two cysteine residues that gate the protein (Umbach et al., 2006). *In vivo*, exogenous GLA application is hypothesized to lead to increased flux through the glyoxylate cycle and, consequently, the TCA cycle (Hewitt et al., 2020a). The latter leads to increased accumulation of NADH, resulting in increased flux of electrons through the cytochrome c (CYTc) pathway and consequent activation of AOX to prevent CYTc overreduction (Calderwood and Kopriva, 2014). (2) Hydrogen sulfide (H_2_S; purple): sulfides accumulate in the cytoplasm as a result of exogenous treatment with H_2_S; these can be converted to the amino acid cysteine (Calderwood and Kopriva, 2014). Cysteine, in turn, may directly modulate the activity of the AOX protein in response to stress or developmental changes in cellular redox state (Jia et al., 2016b; Dhingra and Hendrickson, 2017). (3) Methyl Jasmonate (MeJA; pink): exogenous treatment with MeJA leads to increased flux through the jasmonic acid biosynthesis pathway and accumulation of JA. JA has been shown to activate MYC, a transcription factor (TF) that enhances the function of ethylene signaling factors, particularly EIN/EIL family TFs, leading to an enhanced ethylene response (Shinshi, 2008; Kim J. et al., 2015). It has also been hypothesized that JA can directly influence AOX gene expression, possibly via a similar mechanism, with MYC acting alone or as a complex with other transcription factors to target promoter regions of AOX family genes (Fung et al., 2006). In addition to MYC, JA may regulate additional TFs like RIN, NOR, and cold-induced TFs that target ripening-associated genes (Czapski and Saniewski, 1992; Nham et al., 2017). (4) Cold (red): low temperature causes activation of respiratory burst oxidases (NADPH oxidases) embedded in the cell membrane, which produce superoxide that is then converted to hydrogen peroxide (Marino et al., 2012). Continued stimulation of NADPH oxidases leads to accumulation of ROS in the apoplast; these ROS can then be transferred into the cytosol via aquaporin channels (Qi et al., 2017). When redox state is disrupted, both cytosolic ROS and ROS produced in the mitochondria by the CYTc pathway transmit signals to the nucleus to mediate gene expression changes (including activation of AOX; Li et al., 2013).

While ethylene is important in ripening, the associated hormone perception and signaling pathways do not operate in isolation (McAtee et al., 2013); they may be dependent on other processes or manipulated by altering preripening conditions (Figure 2). Some fruits require a period of cold temperature exposure, known as “conditioning,” in order for ripening to commence (Hartmann et al., 1987). European pear (*Pyrus communis*), for example, must undergo between 15 and 90 days (depending on the cultivar) of conditioning at 0–10°C to activate the S2 autocatalytic ethylene biosynthesis (Villalobos-Acuna and Mitcham, 2008; Hendrickson et al., 2019; Figure 1). Other fruits do not require conditioning to ripen but can be induced to ripen more quickly through appropriate cold storage regimes. Kiwifruit subjected to cold conditioning, upon transfer to room temperature, respires faster, and softens appreciably in comparison to fruits subjected to ethylene preconditioning alone (Ritenour et al., 1999). In ‘Hass’ avocado, for example, exposure to moderate chilling temperatures of 5–10°C for 2 weeks results in the early onset of the respiratory climacteric by 2–7 days in comparison with control fruits stored at 20°C, without any negative impacts. However, longer storage and colder storage temperatures result in a reduced climacteric response and development of symptoms of chilling injury in avocado (Eaks, 1983).

Recent independent studies, as well as reviews, have indicated that such modifications to the classical climacteric ripening implicate several pathways that operate concomitantly with ethylene biosynthesis and perception, including AOX respiration, signaling by reactive oxygen species (ROS), and pathways that may be triggered by changes in epigenetic signatures (Perotti et al., 2014; Kumar et al., 2016; Farinati et al., 2017; Bucher et al., 2018; Hendrickson et al., 2019; Hewitt et al., 2020b). New advances in genome editing have provided further insight regarding potential sites for variation in ripening, as manipulation of upstream transcriptional regulators (Ito et al., 2017) leads to alterations in fruit texture, photoperiodic response, and posttranscriptional regulation of ripening-related genetic elements (Martín-Pizarro and Posé, 2018). Understanding the way in which other key pathways may interact with ethylene biosynthesis and response during ripening will lend important insight into how control of ripening in various fruits can be fine tuned to increase predictability and marketability.

### A Novel Role for AOX in Fruit Ripening

The hallmark aspect of climacteric ripening is the respiratory rise that occurs prior to the S1–S2 ethylene transition, in which an initially gradual increase in carbon dioxide evolution is followed by a heightened burst in respiratory activity during the ripening climacteric (Hiwasa-Tanase and Ezura, 2014; Colombié et al., 2017). The respiratory climacteric has been extensively documented in terms of physiology and biochemistry in a number of fruits (Hulme et al., 1963; Krishnamurthy and Subramanyam, 1970; Salminen and Young, 1975; Hartmann et al., 1987; Andrews, 1995; Hadfield et al., 1995). While early studies were foundational to the understanding and characterization of climacteric ripening with respect to total respiration, greater examination of the genetic and biochemical underpinnings of the respiratory climacteric in a variety of systems is needed.

Climacteric respiration represents the combined activity of several mitochondrial pathways that differentially direct electron transport, leading to several possible energetic fates. The first is the cytochrome c (CYTc) pathway. CYTc operates as a result of a proton gradient generated in the mitochondrial intermembrane space and concludes in the production of cellular energy currency via ATP synthase. In plants, CYTc activity also facilitates cellular detoxification via the synthesis of antioxidant compounds, namely, ascorbic acid (Welchen and Gonzalez, 2016; Fenech et al., 2019). The final step in ascorbic acid production is catalyzed by l-galactono-1,4-lactone dehydrogenase (GLDH), an enzyme that also plays a role in the assembly of respiratory complex I and serves as an electron donor to CYTc in the synthesis of ascorbic acid (Millar et al., 2003; Schimmeyer et al., 2016; Welchen and Gonzalez, 2016). Thus, when antioxidant production is high, electron flux through the CYTc pathway is correspondingly increased. Furthermore, when flux through the CYTc pathway is at maximum capacity due to high cellular respiratory demands, the alternative oxidase (AOX) pathway provides a secondary avenue for electron flux, thereby preventing overreduction of the mitochondrial electron transport chain. Unlike CYTc, AOX is insensitive to cyanide-containing compounds, allowing for viability when normal respiratory activity is inhibited (Wagner and Moore, 1997; Rogov and Zvyagilskaya, 2015).

Additionally, expression of AOX can also be modulated via retrograde mitochondrial signaling in response to reactive oxygen species (ROS) or metabolic disruption (Dojcinovic et al., 2005; Li et al., 2013). Because of this, AOX expression has been used both as an indicator of stress and as a metric to infer the energetic and metabolic status of plant biological systems during development (Saha et al., 2016). In almost all plants, AOX proteins are encoded by a small nuclear multigene family, which consists of two gene subfamilies: AOX1 and AOX2 (Polidoros et al., 2009).

The transition from S1 to S2 ethylene biosynthesis involves numerous metabolic changes, which may occur simultaneously or in series, that require a great deal of regulation and feedback mechanisms to ensure that ripening occurs properly. There is increasing evidence, including respiratory partitioning studies, supporting a role of AOX in the modulation of respiration at various stages around the time of climacteric via induction of S2 ethylene, which thereby influences the development of ripening-associated phenotypes downstream (Figure 1; Considine et al., 2001; Xu et al., 2012; Ng et al., 2014; Perotti et al., 2014; Hendrickson et al., 2019).

### Activation of AOX During Ripening

In several fruits, AOX expression and/or activity has been characterized at the preclimacteric and climacteric stages of fruit development and ripening (Figure 1). Banana, a comparatively fast-ripening fruit, displays elevated preclimacteric expression of AOX at the mature green stage (Woodward et al., 2009), while in papaya, AOX expression peaks between the onset of S2 ethylene and the climacteric peak. In tomato and apple, expression reaches a maximum around the same time as the climacteric peak, indicating that the climacteric rise in these fruits can be partially attributed to the increased capacity for mitochondrial oxidation (Duque and Arrabaça, 1999; Xu et al., 2012; Oliveira et al., 2015). In mango, interestingly, AOX peaks after the climacteric, contributing to oxidation during fruit senescence rather than ripening (Considine et al., 2001).

Furthermore, in some fruits, both climacteric and nonclimacteric, AOX activation can be achieved via cold temperature or chemical stimulation (Figure 2). In zucchini and sweet pepper, cold-induced activation of AOX results in the mitigation of chilling injury (Aghdam, 2013; Hu et al., 2014; Carvajal et al., 2015). Recently, it was demonstrated that completion of cold conditioning, facilitating the S1–S2 transition, in European pear coincides with preclimacteric maxima in AOX transcript accumulation (Hendrickson et al., 2019; Dhingra et al., 2020; Hewitt et al., 2020b).

In a subsequent study, chemical genomics approaches to further target AOX in pear fruit led to the discovery of glyoxylic acid (GLA) as a chemical activator of both AOX and ripening (Dhingra et al., 2020; Hewitt et al., 2020a). GLA has been shown to directly activate AOX *in vivo* via interaction with the two cysteine residues that gate the protein (Umbach et al., 2006). Furthermore, transcriptomic characterization of expressed genes in response to GLA implicates the AOX pathway and glyoxylate cycle in a more extensive ripening network wherein exogenous GLA application results in increased flux through the glyoxylate cycle and TCA cycles, the latter of which leads to accumulation of NADH (Hewitt et al., 2020a). This, in turn, results in increased flux of electrons through the CYTc pathway and consequent activation of AOX to prevent CYTc overreduction (Figure 2; Hewitt et al., 2020a,b). In addition to GLA, there is evidence for activation and differential regulation of AOX protein isoforms by TCA cycle intermediates (Selinski et al., 2018). The results of these studies provide information necessary to develop and test “cocktails” of TCA/GLA cycle metabolites that could result in optimal activation of alternative respiration in the context of fruit ripening regulation when applied exogenously to preclimacteric fruit postharvest.

Hydrogen sulfide (H_2_S), though a known phytotoxin, in minuscule doses can enhance alternative pathway respiration and inhibit ROS production (Hu et al., 2012; Luo et al., 2015; Li et al., 2016; Ziogas et al., 2018). Exogenous treatment with H_2_S leads to the accumulation of sulfides in the cytoplasm, some of which are directly converted to the amino acid cysteine (Calderwood and Kopriva, 2014). Cysteine, in turn, may directly modulate the activity of the AOX protein in response to stress or developmental changes in cellular redox state (Jia et al., 2016b). Additional studies have demonstrated the activation of AOX and ripening in response to H_2_S treatment. In ‘Bartlett’ and ‘D’Anjou’ pears, H_2_S facilitated a bypass of normal cold conditioning requirements for ripening, and treated fruit demonstrated increased ethylene evolution, heightened respiratory rate, and activated *AOX* expression (Dhingra and Hendrickson, 2017). In hawthorn fruit, H_2_S application mitigated chilling injury, which is linked to increased AOX activity and antioxidant capacity (Aghdam et al., 2018).

Application of metabolism-regulatory hormones methyl salicylate (MeSA) and methyl jasmonate (MeJA) results in increased expression of AOX, correlating with a reduction in chilling injury in sweet pepper. JA is a known activator of MYC, a transcription factor that enhances the function of ethylene signaling factors, particularly within the EIN/EIL family, thus leading to enhanced ethylene responses (Shinshi, 2008; Kim J. et al., 2015). It has also been hypothesized that JA can directly influence *AOX* gene expression (Fung et al., 2006) via a similar mechanism, with MYC acting alone or in complex with other transcription factors to target promoter regions of *AOX* family genes (Figure 2). In addition to MYC, JA may regulate additional TFs like RIN, NOR, and cold-induced TFs that target ripening-associated genes (Czapski and Saniewski, 1992; Nham et al., 2017; Li et al., 2018).

Together, these findings reveal interesting insights into chemical and hormonal events that operate in an ethylene-independent space during climacteric ripening, as well as how ripening can be better regulated as a result of this information. Novel discoveries presented in recent studies, which complement and expand upon the foundational knowledge of the role of AOX in ripening, have laid the framework for several exciting hypotheses. First, knowledge of how AOX expression can be induced may provide an avenue for development and testing of ripening strategies in fruits whose respiratory profiles are affected by temperature. Furthermore, knowledge of how temperature preconditioning may mitigate chilling injury via AOX stimulation could allow for improved management practices of papaya, avocado, banana, mango, zucchini, and other temperature-sensitive fruits during storage (Lederman et al., 1997; Aghdam, 2013; Carvajal et al., 2015; Luo et al., 2015; Valenzuela et al., 2017).

### Molecular and Metabolic Links Between Respiration and Ethylene

It is clear, based on the simultaneity and interdependency of responses, that respiration and ethylene are physiologically correlated during climacteric ripening—climacteric rise in respiration is accompanied by a spike in autocatalytic ethylene production, and blocking ethylene perception prevents respiration from increasing further (Hiwasa-Tanase and Ezura, 2014; Watkins, 2015). While elucidating the precise connections between the two pathways will require more genetic and metabolic work, the results of several studies point toward crosstalk between ethylene and respiration and implicate AOX expression and signaling by ROS in this connection (Sewelam et al., 2016).

In tomato, 1-MCP treatment reduces transcript levels of *AOX1a* (Xu et al., 2012); the simultaneous inhibition of ethylene response and maintenance of respiration at low levels by 1-MCP indicates a relationship between ethylene, the respiratory climacteric, and AOX at the molecular level. The activity of AOX and biosynthesis of ethylene are directly dependent upon flux through CYTc and the availability of ATP. RNA interference studies in tomato reveal a modulatory role of *AOX* in ethylene production, as ACS4 activity in *AOX-RNAi* plants is significantly lower than in wildtype (WT) plants (Xu et al., 2012). Reduced activity of ethylene biosynthetic enzymes when *AOX* is silenced could be due to a decrease in precursors for ethylene production. For example, the methionine cycle, and therefore ethylene biosynthesis, is dependent upon ATP generation via respiration (Mattoo and White, 2018). Specifically, methionine is converted to the immediate precursor to ACC, SAM, in an ATP-dependent reaction catalyzed by SAM synthetase (Figure 2; Yang and Hoffman, 1984). During ripening, AOX could allow for heightened carbon flux through glycolysis and the TCA cycle; this would prevent overreduction of the ubiquinone pool, increase oxidation of NADH, and accelerate carbon turnover, resulting in the production of large amounts of ATP that could be used for S2 ethylene and other ripening-associated metabolic processes. Moreover, a recent report of a link between the TCA and methionine metabolism (and consequently, ethylene metabolism) via NADH oxidation lends further support to this concept (Lozoya et al., 2018).

In cucumber, brassinosteroids were reported to induce ethylene responses and ROS, the collective activities of which resulted in stimulation of AOX and activation of downstream abiotic stress responses (Wei et al., 2015). ROS, which are produced in the mitochondria during respiration and accumulate in the apoplast as a result of abiotic stimulation of respiratory burst oxidase (NADPH oxidase) homologs, were historically thought of in terms of their toxicity to plants in high concentrations; however, their critical roles in response to perturbations in cellular redox state have become clearer in recent years (Jimenez et al., 2002; Marino et al., 2012; Vaahtera et al., 2013; El-Maarouf-Bouteau et al., 2015; Kumar et al., 2016; Noctor et al., 2018; Decros et al., 2019). During fruit maturation, ROS accumulation peaks once at the start of ripening (presumably the start of the respiratory climacteric) and again at overripening, around the time of harvest maturity (Muñoz and Munné-Bosch, 2018). This accumulation may coincide with the activation of AOX in certain species (Figure 1). In *Arabidopsis*, ethylene-induced signaling by hydrogen peroxide (H_2_O_2_), a form of ROS, was shown to activate AOX in response to cold temperatures (Wang et al., 2010, 2012). It has been suggested that such temperature-induced transcriptional changes in AOX occur via ROS derived from NADPH oxidase activity (Vanlerberghe, 2013; McDonald and Vanlerberghe, 2018). These NADPH oxidases produce O_2_^−^, which is converted to H_2_O_2_, in the apoplast. These ROS are translocated to the cytoplasm via aquaporin channels (Qi et al., 2017). The influx of apoplastic ROS, along with additional species produced in the mitochondria, serves to activate nuclear-targeted redox signals, which elicit antioxidative responses and alteration in metabolic processes (Suzuki et al., 2011; Marino et al., 2012; McDonald and Vanlerberghe, 2018; Chu-Puga et al., 2019; Figure 2). Such ROS-induced retrograde signaling leads to modulation of AOX expression, as has been shown in potato and pea. It has been hypothesized that in this way, ROS may facilitate crosstalk between respiration and ethylene via AOX activation in fruit ripening (Marti et al., 2009; Hewitt et al., 2020b; Hua et al., 2020).

Taken together, these results indicate that an interplay between different components of several metabolic signaling pathways is responsible for initial AOX activation. Furthermore, the activity of both ethylene and alternative respiratory pathways may be self-perpetuating by means of an autostimulatory feedback loop involving ROS (Wei et al., 2015). While AOX serves as a mechanism to prevent overreduction of the CYTc pathway, it is possible that overstimulation of AOX via external perturbations (e.g., chemical or temperature) in fruit prior to the S2 transition induces a vacuum effect, drawing the glycolytic pathway and TCA cycle into action to deliver more reducing power, thereby initiating CYTc pathway respiratory activity. For example, in *Arabidopsis* and tomato, *aox* mutants displayed disrupted accumulation of primary respiratory metabolites, which affect development (Xu et al., 2012; Jiang et al., 2019). The same may be true of AOX impairment in fruit. Understanding the regulation of the respiratory climacteric and how crosstalk between ethylene and AOX is facilitated may require a look at the transcriptional regulators of these responses.

### Transcriptional Regulation Modulates Both Ethylene and Respiratory Responses

Within the last decade, the importance of transcriptional regulation of ripening response has become more evident. During ripening, signals from upstream transcription factors (which may be activated by environmental or intrinsic triggers) facilitate a cascade of downstream signaling activity (Cherian et al., 2014; Gao et al., 2020). In fruits, this signaling activity leads to increased respiration, cell wall softening, and changes in the production of pigments, volatiles, starch, and sugar content, and phytonutrient metabolite content—these processes are all characteristic of ripening, with respiration and biosynthesis of ethylene particularly relevant to climacteric ripening (Seymour et al., 2013a; Karlova et al., 2014). Among some of the most important transcriptional regulators during ripening are ripening inhibitor (RIN), colorless non-ripening (CNR), tomato Agamous 1 (TAG1), tomato Agamous-like 1 (TAGL1), fruitful 1 and 2 (FUL1 and 2), and non-ripening (NOR); all of these are involved in a complex and interconnected regulatory network that ultimately leads to fruit ripening and the aforementioned ripening-associated qualities (Figure 2; Seymour et al., 2013a; Fujisawa et al., 2014; Datta and Bora, 2019). The availability of mutants targeting the aforementioned ripening regulators in tomato allows for study of consequences of ripening perturbation at the regulatory level. Many studies have demonstrated the detrimental effects of mutations in these key transcriptional regulators on ethylene production and signaling, which are expected to have further downstream respiratory consequences via the avenues for the interpathway crosstalk discussed in the previous section.

Because of its resultant complete inhibition of ripening in tomato, the *rin* mutation has become one of the most iconic ripening-associated mutations in studies of climacteric fruit. *Rin* mutant tomatoes fail to mature beyond the green-ripe stage and do not exhibit the characteristic ripening climacteric of wild-type fruit (Vrebalov et al., 2002). Commercial varieties of tomato, heterozygous for the *rin* mutation, have been introduced to the market and have displayed increased shelf life with little noticeable alteration to the desired flavor profile (Garg et al., 2008). Chromatin immunoprecipitation studies revealed several direct targets of *RIN*, including the ethylene-biosynthesizing enzyme 1-aminocyclopropene carboxylate oxidase 4 (ACO4) and α-galacturonase (α-gal), an enzyme-associated with cell wall breakdown and fruit softening (Martel et al., 2011; Fujisawa et al., 2012). Furthermore, RIN protein binding sites (CArG box) were identified in the promoter regions of *α-gal* and *ACO4*, and *ACS2* genes (Fujisawa et al., 2012, 2013). Binding to these motifs is facilitated by localized demethylation of associated promoter regions (Li et al., 2017). Expression of ethylene biosynthetic enzymes ACS1 and ACO1 in apple was greatly decreased when expression of various *RIN-like* MADS-box genes was downregulated or silenced (Ireland et al., 2013). Bisulfite sequencing studies revealed that binding sites in the promoter regions of known transcriptional targets of RIN were found to be demethylated, suggesting that demethylation is necessary for RIN binding and development (Zhong et al., 2013). Treatment with the methyltransferase inhibitor 5-azacytidine resulted in fruit that ripened prematurely, further lending support to demethylation of binding sites as a trigger for RIN-activated ripening (Zhong et al., 2013; Liu R. et al., 2015). While the *rin* mutant was classically understood as a loss of function mutant, more recent work suggests that it is actually a gain-of-function mutant that produces a protein that actively represses ripening (Ito et al., 2017). Regardless, it is clear that when RIN is perturbed, ripening does not proceed to completion.

Colorless non-ripening (CNR) transcription factor is a squamosal-promoter binding-like protein, which appears to be necessary for RIN to bind to promoters (Martel et al., 2011; Zhong et al., 2013). With a hypermethylated, heritable promoter that results in reduced transcriptional activity, CNR is a unique example of an epiallele, requiring the activity of a specific chromatin-methylating enzyme, chromomethylase3, for somatic inheritance (Ecker, 2013; Seymour et al., 2013b; Chen et al., 2015). Thus, the CNR transcription factor lends evidence for the role of epigenetics in critical developmental transitions such as those that occur during S1–S2 ethylene production and ripening.

TAG1, TAGL1, and MADS-box transcription factors FUL1 and FUL2 can form complexes with RIN (Shima et al., 2013; Fujisawa et al., 2014). Mutation of these regulatory factors results in fruit with decreased ripening capacity or non-ripening phenotypes (Itkin et al., 2009; Garceau et al., 2017). Another TF among the core set of regulatory elements is the NAC-domain-containing protein at the tomato non-ripening (NOR) locus. NOR mutants fail to ripen in a physiologically similar manner to RIN and TAGL1 mutants. NOR acts upstream of ethylene biosynthesis and, like RIN, appears to bind to promoter regions of genes involved in ethylene biosynthesis, thereby positively regulating ripening (Gao et al., 2018; Figure 2). It is unclear whether RIN and NOR interact with one another to stimulate ripening in conjunction. Considering increasing understanding of their regulatory role in ripening, NOR and FUL genes have been recent targets for improving shelf life in tomato fruit (Nguyen and Sim, 2017; Wang et al., 2019).

Recently, the role of AOX has been investigated in NOR, CNR, and RIN mutant fruit (Manning et al., 2006; Xu et al., 2012; Perotti et al., 2014). AOX activity elicits differential effects in each of these mutants, and expression of RIN, CNR, and the ethylene receptor never ripe (NR) has been observed in fruit in which *AOX* is silenced. When *AOX* was inhibited via RNA interference, the expression of these transcriptional regulators decreased (Xu et al., 2012). Because CNR acts upstream of ethylene biosynthesis and the NR receptor acts downstream, this observation suggests that AOX plays a yet uncharacterized role in ripening mediated by transcriptional regulators that affect ethylene biosynthesis, signal transduction, and response (Giovannoni, 2004; Seymour et al., 2013b; Hewitt et al., 2020b). Interestingly, it has been hypothesized that the activation of both NOR and RIN may be linked, either directly or indirectly, to jasmonic acid signaling (Czapski and Saniewski, 1992). As indicated previously, JA is known to enhance transcriptional activation of other ripening-associated processes, including AOX. In addition to JA, the ethylene signaling molecule EIN3/EIL1 is hypothesized to directly activate RIN in tomato, thus serving as an instrumental part of a positive feedback loop resulting in autocatalytic ethylene production (Figure 2; Lü et al., 2018), and corresponding to increased consumption of ATP produced from CYTc respiration.

### Epigenetic Regulation of Ripening and a Potential Link to the Respiratory Climacteric

Epigenetics refers to the heritable modifications of the genome beyond the physical nucleotide sequence, including DNA methylation and modifications to histone proteins. In contrast, epigenomics refers to all modifications, regardless of heritability (Giovannoni et al., 2017). One of the most commonly studied forms of epigenetic modification is DNA methylation. Methylation status is in constant flux due to the changing environment; therefore, condition-specific methylation status may be used to infer stress conditions, ripening competency, and developmental progress, among other things. Recent evidence suggests that perturbation of mitochondrial function has an effect on epigenetics. At the same time, continued oxidation of NADH serves to counteract the increase in nuclear DNA methylation and maintain cellular homeostasis (Lozoya et al., 2018).

The causes of such perturbations may vary. Temperature is known to be a major factor in alteration of methylation status, and conditioning of fruit requiring chilling to ripen, or to avoid chilling injury, could affect the methylation of promoter regions of key ripening related genes and regulatory transcription factors. With more tools for epigenetic and epigenomic analyses available, including bisulfite sequencing and PacBio long-read sequencing, new insights are being gained into the impact of epigenetic signatures on development and senescence of fruit (Daccord et al., 2017; Xu and Roose, 2020). Understanding how the epigenome governs downstream transcriptional regulation and response is critical to better understanding ripening and senescence.

Chemically induced demethylation of tomato fruit using 5-azacytidine results in early ripening of fruit (Zhong et al., 2013). This finding indicates that the alteration of methylation status is one of the first steps in the regulation of downstream processes associated with ripening. Recent transcriptomic and gene ontology enrichment analysis of cold conditioned ‘D’Anjou’ and ‘Bartlett’ pear fruit suggests that both methylation and chromatin modifications may be important for activation of vernalization-associated genes and ripening-associated transcriptional elements, which were activated in conjunction with AOX during prolonged cold temperature exposure (Hewitt et al., 2020b). Interestingly, these two cultivars appear to differentially engage expression of vernalization genes VRN1 and VIN3, which could justify the need for different cold exposure time in different cultivars (Hewitt et al., 2020b). Studying the epigenomic status in light of mutations to key transcription factors, mentioned previously, illuminates the way that DNA methylation and histone modifications in genetic regulatory elements serve to modulate certain aspects of ripening early on in development. Beyond abiotic influences, it is possible that some signal from mature seeds is originally what signals the onset of ripening progression (McAtee et al., 2013). This hypothesis is supported by the recent characterization of enzymes responsible for removing epigenetic signatures to DNA or histones, such as the recently characterized DEMETER-like DNA demethylase gene *SlDML2* in tomato (Liu R. et al., 2015; Zhou et al., 2019). These proteins are particularly highly expressed in the locular tissue surrounding mature seeds in the fruit. In contrast to tomato, a climacteric fruit in which demethylation is an important factor in ripening, nonclimacteric orange fruit was recently reported to exhibit global increases in DNA methylation as ripening progresses (Huang and Liu, 2019). In addition to factors known to directly affect methylation, recent evidence suggests that additional factors, including noncoding RNAs, circular RNAs, and microRNAs, may target genes with specific methylation patterns or abundance to elicit changes in expression (Zuo et al., 2020). The breadth of factors that may contribute to alterations in the epigenome is still being elucidated; however, it is possible that methylation status during ripening is tied to fruits’ classifications as climacteric or nonclimacteric. This, in turn, is expected to differentially impact a wide range of ripening-associated parameters, in addition to ethylene and respiratory profiles.

## Conclusions and Future Perspectives

The interplay of ethylene (biosynthesis, signaling, and response) and respiration (CYTc and AOX) have been extensively characterized at the physiological level. Studies conducted within the last several years provide new insights with respect to the connection between these two critical pathways at the molecular and metabolic levels. AOX activity begins to increase during the preclimacteric phase, prior to the S1–S2 ethylene transition, particularly in the context of cold temperature or other external stimuli. This activity may be accompanied by the accumulation of ROS as the CYTc pathway capacity reaches a maximum. At the onset of S2 ethylene stimulation, both ethylene response and alternative pathway respiration appear to be interdependent. Ethylene biosynthesis requires ATP, generated via respiration, and activity of respiratory pathways modulated by ERFs in the nucleus; this is evidenced by the inhibition of ethylene production and respiration by the ethylene receptor antagonist 1-MCP. The commencement of both ethylene and respiration-associated processes is likely due to upstream transcriptional and epigenetic regulators, as well as the signaling activity of ROS generated during increased respiration. Mutants of master transcriptional regulators in tomato have provided a means for the study of their effects on both ethylene production and respiration during ripening. Furthermore, studies have investigated the effects of temperature and chemical manipulation of AOX with the aim to understand ways in which the timing of ripening can be controlled. AOX and related processes represent a new frontier in regulating postharvest ripening and, therefore, better management to reduce fruit wastage and development of postharvest management strategies in different types of fruits.

The ever-growing fields of genomics, transcriptomics, metabolomics, and epigenomics offer high-throughput strategies for extrapolation of important biological function and expression information from large datasets generated in recent ripening experiments (Morozova and Marra, 2008; Banerjee et al., 2019). The relatively new ability to examine plant epigenomes, and most recently the “ripenome” at the level of single nucleotide bases reveals further avenues for understanding epigenetic regulators of the transition from preclimacteric to climacteric, and the subsequent development of species-specific ripening phenotypes (Giovannoni et al., 2017). Advances in gene editing have provided improved tools by which candidate ripening-associated genes, identified using the aforementioned “omics” approaches, can be targeted in a concise way to achieve specific results with limited or no off-target effects. Use of these new approaches will facilitate improved understanding of the array of diverse transcriptional responses, the interaction of associated pathways (including AOX, vernalization-associated, and organic acid metabolic pathways), functional implications of the S1–S2 transition, and interdependent roles of ethylene and respiration in ripening (Nham et al., 2017; Hewitt et al., 2020a,b). Such approaches used independently or in conjunction will facilitate the identification and targeted manipulation of candidate regulators of important ripening and fruit quality-associated characteristics in fruits. This, in turn, could translate to greater storability of fruits after harvest, improved marketability within respective fruit industries, and enhanced consumer satisfaction overall.

## Author Contributions

SH and AD conceptualized the review. Both the authors contributed to the article and approved the submitted version.

### Conflict of Interest

The authors declare that the research was conducted in the absence of any commercial or financial relationships that could be construed as a potential conflict of interest.
